# Cerium-Containing N-Acetyl-6-Aminohexanoic Acid Formulation Accelerates Wound Reparation in Diabetic Animals

**DOI:** 10.3390/biom11060834

**Published:** 2021-06-03

**Authors:** Ekaterina Blinova, Dmitry Pakhomov, Denis Shimanovsky, Marina Kilmyashkina, Yan Mazov, Tatiana Demura, Vladimir Drozdov, Dmitry Blinov, Olga Deryabina, Elena Samishina, Aleksandra Butenko, Sofia Skachilova, Alexey Sokolov, Olga Vasilkina, Bashar A. Alkhatatneh, Olga Vavilova, Andrey Sukhov, Daniil Shmatok, Ilya Sorokvasha, Oxana Tumutolova, Elena Lobanova

**Affiliations:** 1 Department of Clinical Anatomy and Operative Surgery, Department of Pathological Anatomy, Institute for Regenerative Medicine, Sechenov University, 8/1 Trubetzkaya Street, 119991 Moscow, Russia; bev-sechenov@mail.ru (E.B.); denisshimmm@mail.ru (D.S.); yan.med@mail.ru (Y.M.); demura-t@yandex.ru (T.D.); vndrozdov@yandex.ru (V.D.); butenko.aleksa@mail.ru (A.B.); dr.alex.sokolov@gmail.com (A.S.); olga.vavilova18@yandex.ru (O.V.); center.skin@gmail.com (A.S.); 2 Department of Morphology, National Research Nuclear University MEPHI, 31 Kashirskoe Highway, 115409 Moscow, Russia; 3 Laboratory of Pharmacology, Department of Pathology, National Research Ogarev Mordovia State University, 68 Bolshevistskaya Street, 430005 Saransk, Russia; diman0910@mail.ru (D.P.); marina.kilmyashkina.1998@mail.ru (M.K.); dr.deryabina@gmail.com (O.D.); ms.vasilkina@bk.ru (O.V.); d.bashar81@mail.ru (B.A.A.); masonry_25@mail.ru (D.S.); tumutol@rambler.ru (O.T.); 4 Laboratory of Molecular Pharmacology and Drug Design, Department of Pharmaceutical Chemistry, All-Union Research Center for Biological Active Compounds Safety, 23 Kirova Street, 142450 Staraya Kupavna, Russia; samy-elena@yandex.ru (E.S.); skachilova@mail.ru (S.S.); sorokvasha@gmail.com (I.S.); 5 Department of Pharmacology, A.I. Yevdokimov Moscow State University of Medicine and Dentistry, 20/1 Delegatskaya Street, 127473 Moscow, Russia; e.g.lobanova@mail.ru

**Keywords:** cerium-containing formulation, diabetic wound, toxicity, proliferation, micro-vessels, anti-microbial activity, topical application, cytokines, inflammation

## Abstract

Background: The main goal of our study was to explore the wound-healing property of a novel cerium-containing N-acethyl-6-aminohexanoate acid compound and determine key molecular targets of the compound mode of action in diabetic animals. Methods: Cerium N-acetyl-6-aminohexanoate (laboratory name LHT-8-17) as a 10 mg/mL aquatic spray was used as wound experimental topical therapy. LHT-8-17 toxicity was assessed in human skin epidermal cell culture using (4,5-dimethylthiazol-2-yl)-2,5-diphenyltetrazolium bromide (MTT) assay. A linear wound was reproduced in 18 outbred white rats with streptozotocin-induced (60 mg/kg i.p.) diabetes; planar cutaneous defect was modelled in 60 C57Bl_6_ mice with streptozotocin-induced (200 mg/kg i.p.) diabetes and 90 diabetic *db*/*db* mice. Firmness of the forming scar was assessed mechanically. Skin defect covering was histologically evaluated on days 5, 10, 15, and 20. Tissue TNF-α, IL-1β and IL-10 levels were determined by quantitative ELISA. Oxidative stress activity was detected by Fe-induced chemiluminescence. Ki-67 expression and CD34 cell positivity were assessed using immunohistochemistry. *FGFR3* gene expression was detected by real-time PCR. LHT-8-17 anti-microbial potency was assessed in wound tissues contaminated by MRSA. Results: LHT-8-17 4 mg twice daily accelerated linear and planar wound healing in animals with type 1 and type 2 diabetes. The formulated topical application depressed tissue TNF-α, IL-1β, and oxidative reaction activity along with sustaining both the IL-10 concentration and antioxidant capacity. LHT-8-17 induced Ki-67 positivity of fibroblasts and pro-keratinocytes, upregulated *FGFR3* gene expression, and increased tissue vascularization. The formulation possessed anti-microbial properties. Conclusions: The obtained results allow us to consider the formulation as a promising pharmacological agent for diabetic wound topical treatment.

## 1. Introduction

A therapeutic approach to diabetic wound healing is one of the most challenging problems nowadays [1]. Worldwide, diabetic foot ulcer as a most common diabetes mellitus (DM) complication has a deteriorating impact on humans and society due to quality of life stagnation, a high rate of disability, and financial and economic burdens. Epidemiological studies report about a 25% life-time risk of foot ulceration in the diabetic population with approximately 24% of all health care expenses related to diabetic foot complications [2,3,4].

Disturbances of skin integrity may occur as an outcome of the general disease course, traumatic injury or surgical manipulations. The type of metabolic disorder also plays a key role in diabetic wound development and progression [5]. Delay and difficulties in diabetic wound reparation are strongly associated with molecular, cellular, and tissue changes at different stages of the healing process. They involve particularities of inflammatory regulation, vascularization of newly-developing tissues, and cellular differentiation and growth [6,7,8]. Molecular signaling and cellular cooperation limit the timing of healing completion, orchestrate the direction of reparation (substitution or restitution), and determine both the structure and stability of the forming scar [7,8]. Bacterial contamination of skin defects advances the damage severity that needs additional intervention and care [9]. Hence, current treatment options for diabetic wound healing require multidisciplinary solutions including surgery and non-surgical applications. Topical application of pharmacological agents or formulation is used to treat inflammation, for reparation, and to prevent microbial contamination.

Cerium, a metal of the lanthanoid group, possesses a broad range of pharmacological properties [10,11]. As an oxide and an organic salt, it demonstrates antimicrobial and regenerative activity, especially in burning soft tissue damage [12,13,14]. Recent studies demonstrated the high potency of metal-containing compounds to control oxidative stress and modulate neurotransmission [15,16]. N-Acethyl-6-aminohexanoic (ε-aminocaproic) acid has long been known for its antifibrinolytic activity [17]. Serkedjieva et al. showed the antimicrobial and antiviral property of a novel compound based on ε-aminocaproic acid. Some recent investigations reported the acid anti-inflammatory potency [18]. A novel formulation of the cerium-containing salt of N-acethyl-6-aminohexanoic acid has been synthetized in the Department of Pharmacology and Drug Design of the All-Russia Scientific Center of Biological Active Compounds Safety. The main goal of our study was to explore the wound-healing property of the novel cerium-containing N-acethyl-6-aminohexanoate acid compound and determine key molecular targets of the compound mode of action in diabetic animals.

## 2. Materials and Methods

### 2.1. Ethic Statement

All experiments with laboratory animals were carried out in accordance with the European Convention for the Protection of Vertebrates Animals used for Experimental and Other Scientific Purposes regulations. The study protocol was reviewed and approved by Sechenov University Ethic Committee at the meeting on 17 September 2020 (Rev. No. 115/09/17).

### 2.2. Pharmacological Substance and Reference Medications

We explored the wound-healing property of the novel compound, cerium N-acetyl-6-aminohexanoate (laboratory name LHT-8-17), as a pharmaceutical substance containing nanoparticles with 99.78% purity, which was designed and synthetized in the Department of Pharmacology and Drug Design of the All-Russia Scientific Center of Biological Active Compounds Safety (ARSC BAC, Russia). The compound was used as a 10 mg/mL colloid solution in the form of a spray with pH 7.3. For solubilization, we used a water–propylene glycol mixture. One dose of the formulation contained 2 mg of the active substance in 0.2 mL of the spray. We used D-panthenol (250 mL, purity > 98.0%, Merck, Darmstadt, Germany) in the form of an aqueous spray as a comparative wound-healing pharmacological agent. Several studies reported safe and effective use of D-panthenol as a constituent of cosmetics and active component of wound dressings and aerosols at a range of concentration from 0.1% to 5.0% [19,20,21,22,23]. As the dose-dependent wound healing potency of the molecule has not been investigated, we used the same D-panthenol-containing spray concentration as for the studied agent. One dose of D-panthenol also contained 2 mg of the active substance in 0.2 mL of the spray.

### 2.3. Cellular Toxicity Assay

In vitro LHT-8-17 cytotoxicity was assessed by the reduction of epidermal cells’ metabolic activity as previously described [24]. The human skin epidermal cell line (HaCaT) was purchased from CLS Cell Lines Service (Heidelberg, Germany). Cells at 1 × 10^4^ cells/well density were seeded in 96-well plates and then incubated with different concentrations (0, 15, 30, 45, 60, 75, and 90 µg/mL) of LHT-8-17 dissolved in PBS. After 24 h of exposure, culture media were substituted with 0.5 mg/mL (4,5-dimethylthiazol-2-yl)-2,5-diphenyltetrazolium bromide-containing media (MTT, Merck, Sigma–Aldrich, Darmstadt, Germany) and incubated for 4 h at 37 °C. To dissolve forming crystals of formazan produced by survived cells, we used dimethyl sulfoxide. Optical transparency was measured and analyzed for each plate at a 530 nm wavelength using a microplate semi-automatic reader i.e., Statfax-4200 (Awareness Technology, Palm City, FL, USA). Cell viability was presented as a percentage of the appropriate control as the median and SD.

### 2.4. Laboratory Animals and Models of Experimental Diabetes Mellitus, Pain Management

Two experimental approaches to DM modelling were used. Type 1 DM (T1D) was induced by single administration of streptozotocin (substance with purity > 98.0%, Merck, Darmstadt, Germany), 60 mg/kg intraperitoneally (i.p.), in outbred white laboratory rats of both sexes weighing 200–220 g, and 200 mg/kg i.p. in C57Bl_6_ mice of both sexes weighing 18–20 g and fasting 24 h before and after the injection [5]. To control the pathology onset, the serum glucose level was registered 48 h after diabetogenic agent administration using an automated biochemical analyzer FUJI DRI-CHEM NX500i (FUJIFILM, Tokyo, Japan). As a model of type 2 DM (T2D), we used 8-week-old diabetic *db*/*db* mice strain C57BLKS/J of both sexes [25]. Laboratory animals were purchased from the specific pathogen-free (SPF) Laboratory Animals Breeding Facility of the Shemiakin and Ovchinnikov Institution of Bioorganic Chemistry of Russian Academy of Sciences (Moscow, Russia). The animals were kept under natural daylight conditions in separate cages with 55–60% humidity and 18–22 °C room temperature. Mice with infected wounds were isolated in sterile separately ventilated cages immediately after a pathogen inoculation.

We used the facial mimic scale for evaluation of postoperative pain reaction [26]. Moderate pain was managed by intragastric administration of 100 mg/kg ketoprofen (substance K1751 with purity > 98.0%, Merck, Darmstadt, Germany) two times a day [27].

### 2.5. Wound Modelling and Experimental Groups

A 30 mm linear wound was reproduced in 18 anesthetized (thiopental-sodium, SANDOZ, Kundl, Austria, 40 mg/kg i.p.) laboratory outbred rats, nine males and nine females, with streptozotocin-induced T1D on day 10 of the pathology onset according to [28]. Depilated skin of the animal’s back was dissected to the muscular layer along the spine by a sterile blade and then stitched by a 2-0 sterilized catgut surgical suture (Ethicon, Somerville, NJ, USA) sparing 10 mm between neighboring stitches. The wounded animals were randomly divided into three groups with six rats, three males and three females, in each group: control, LHT-8-17, and D-panthenol. Experimental wounds were sprayed by 0.4 mL of phosphate-buffered saline solution (PBS), the same volume of LHT-8-17 (4 mg of the substance per one application), and D-panthenol (4 mg of the substance per one application) twice daily for 14 days. On day 14, stitches were removed, and three weeks after the wound modelling, laboratory rats were euthanized; 40 × 20 mm^2^ skin slopes were dissected. On day 21 of the observation (seven days after experimental topical treatment cessation), the scar strength was measured using a self-modified isometric force system (Ugo Basile, Gemonio, Italy).

A planar wound was modelled in both streptozotocin-premedicated C57Bl_6_ mice and T2D *db*/*db* mice under 40 mg/kg thiopental-sodium i.p. anesthesia according to [29]. Depilated skin slope in the withers area, 15 × 15 mm^2^, was surgically removed under sterile conditions, and the wound was left uncovered during the survey period. Wounded animals of each line were randomly assigned to control, LHT-8-17, and D-panthenol groups (n = 20 in each group). Experimental wounds were sprayed twice daily from day 1 for 20 days with PBS, LHT-8-17, and D-panthenol as described above. The surgery was followed by the wound daily calipering as well as measurement of the postoperative scar area on the day when the skin defect showed complete closure. It was defined as a hairless fibrous area, which formed as an result of skin wound healing. Five, ten, fifteen, and thirty days after the wound modelling, five animals in each group were randomly allocated and euthanized; wound tissues were removed and divided into two equal pieces for further processing. Therefore, the number of animals in each study group presented the total number of mice excluded gradually from the survey at its four stages. At each point of molecular, histological, and biochemical analyses, the study subgroups included no more than five mice for each kind of intervention. Tissue particles for morphological and microbiological examination and biochemistry were processed immediately upon removal, while the others for immunohistochemistry (IHC), enzyme-linked immunoassay analysis (ELISA), and molecular analysis were frozen.

An infected wound was reproduced as the planar one contaminated by 10 μL of fresh culture of methicillin-resistant *Staphylococcus aureus* (*S. aureus*) АТСС 43300 (Culti-Loop, OXOID, Thermo-Fisher Scientific, Waltham, MA, USA), 1.5 × 10^8^ colony forming units (CFU) in Mueller-Hinton broth (BD, USA), in a sterile box. Thirty T2D *db*/*db* mice with freshly-made contaminated wounds were randomly designated to three groups (n = 10 in each group) according to the topical treatment option. Antimicrobial efficacy of LHT-8-17 was compared with the control (PBS) and 0.05% chlorhexidine (Chlorhexidine digluconate, substance with purity > 98.0%, Chemical Systems, Moscow, Russia) as sterile sprays [30,31].

### 2.6. Morphological Examination

Wound tissues were removed, fixed in 10% phosphate-buffered formalin, and processed automatically using the STP-120 (Thermo-Fisher Scientific, Waltham, MA, USA) histology station. Paraffin-embedded tissues were cut by a microtome, i.e., НМ340Е (Microm Laborgerate GmbH, Berlin, Germany). Four-mm-thick frontal sections were stained by hematoxylin and eosin and viewed under a light microscope, OLYMPUS BX51 (OLYMPUS, Tokyo, Japan). We also used Van Geison’s staining to assess collagen production. The severity of the inflammatory reaction, number of fibroblasts and fat cells, and level of epithelization were examined using the Shekhter et al. histological score table [32].

### 2.7. Immunohistochemistry (IHC)

СD34 positivity of endothelial cell membranes and Ki-67 cell expression were evaluated by immunohistochemistry (IHC). We used IHC with a heat-induced epitope retriever (Thermo Fisher Scientific, USA). The fresh material of excised wounds was fixed in 10% neutral formalin and embedded in paraffin. Four millimeter sections were made and fixed on slides in 24 h at 37 °C using an incubator—Ecross PE-4312 (Ecross, Moscow, Russia). Only one slide from each case was stained by hematoxylin and eosin, and the other four were kept for further IHC analysis as per the manufacturer’s protocol. Two slides from each case were stained by primary antibodies, and the other two were positive and negative controls. We used recombinant rabbit monoclonal anti-CD34 antibody (clone [EP373Y], Abcam, Cambridge, UK) and Ki-67 rabbit monoclonal antibody (clone [SP6], Abcam, Cambridge, UK). Rat kidney and spleen tissues were used for CD34 and Ki-67 positive controls, respectively. Negative controls were the slides stained by the same protocol but without primary antibodies.

CD34 expression was estimated as the number of vessels per two randomly chosen high-power fields (100×) capturing fresh granulation tissues within 1000 μm of the wound bottom central point. The results were scored by two independent pathologists who were blinded to the study group characteristics, type of topical treatment, and timing of autopsy. In cases (n = 4) where scores determined by the two observers differed, the specimens were reassessed to reach consensus. Ki-67 expression assessment in wounds was scored as a percentage of cell nuclei positivity in five randomly chosen fields capturing all layers of the wound bed (400×).

### 2.8. Enzyme-Linked Immunosorbent Assay (ELISA)

We used sandwich enzyme-linked immunosorbent assay (ELISA) and murine TNF-α, IL-1β, and IL-10 ELISA kits (CUSABIO BIOTECH Co. LTD, Wuhan, China) to assess the tissue cytokine concentration on day 5 of the wound reparation. In brief, fresh granulations were consequently frozen (−20 °C) and thawed trice, and then the homogenate was centrifuged (5× *g* for 5 min) at 2–8 °C. The supernatant was pipetted in a 96-well microplate with monoclonal anti-TNF-α, anti-IL-1β or anti-IL-10 antibodies. After washing out, unbound components were removed, and a conjugate of polyclonal antibodies to TNF-α, IL-1β or IL-10 with biotin was added to each well. Then, avidin—horseradish peroxidase complex was added to each well for the reaction enhansement. After the TMB chromogenic reaction was stopped, optical transparency was measured and analyzed for each plate at 652 nm on a StatFax 4200 semi-automatic reader (Awareness Technology, Palm City, FL, USA).

### 2.9. Tissue Oxidative Stress Assay

Local oxidative stress activity (LOSA) and total antioxidant capacity (TAC) in homogenates of connective tissue from wound beds of diabetic mice were assessed on days 5 and 10 by the Fe-induced chemiluminescence method as previously described [33]. Fresh granularions were rinsed in PBS, homogenized under cold temperatures (0–4 °C) with PBS, 0.5 mL per 100 mg, and centrifuged at 3000× *g* for 15 min. Then, 0.02 mL of 2% hidrogenim peroxide was added to a cuvette containing 0.3 mL of the supernatant with 0.5 mL of phosphate buffer (pH 7.5), with subsequent measurement of base chemiluminescense using SmartLum-1200 (DISoft, Moscow, Russia). The reaction was activated by 1 mL of 0.05 mM Fe(II) sulfate. Activated chemiluminescense was detected after 5 min.

### 2.10. FGFR3 Expression Assay

DNA and RNA were isolated from thawed and homogenized particles of freshly-frozen wound tissues using the AllPrep DNA/RNA/miRNA Universal Kit (Qiagen, Hilden, Germany) according to the manufacturer’s recommendations. The nucleic acid concentrations were measured with a Qubit 4 fluorimeter (Thermo Fisher Scientific, Waltham, MA, USA) using Qubit dsDNA HS Assay and Qubit RNA BR Assay kits (Thermo Fisher Scientific, Waltham, MA, USA). For each sample, 100 ng of total RNA was mixed with 20 pm of a reverse transcription primer oligonucleotide (for *FGFR3* and *ACTB* expression, we used random decamers) in 9 µl and incubated at 70 °C for 2 min, and then chilled on ice. Reverse transcription was performed at 42 °C for 30 min using the MMLV RT kit (Evrogen, Moscow, Russia) according to the manufacturer’s recommendations. Reverse transcription was then stopped by inactivating reverse transcriptase. The obtained cDNA was 10-fold diluted and used for RNA expression analyses using real-time PCR experiments.

We performed real-time polymerase chain reaction (PCR) experiments using a DTPrime amplifier (DNATechnology, Moscow, Russia). For *FGFR3* and *ACTB* expression, qPCRmix-HS SYBR (Evrogen, Russia) was used according to the manufacturer’s recommendations. The following oligonucleotide primers were used for *FGFR3*: *FGFR3RT*-*F*, 5′-*CCCAAATGGGAGCTGTCTCG*-3′; *FGFR3RT*-*R,* 5′-*CATCTCAGACACCAGGTCCG*-3; for *ACTB: b-act-for,* 5′*-GAGCGGGAAATCGTGCGTGACATT*-3′; *b-act-rev*; 5′-*GATGGAGTTGAAGGTAGTTTCGTG*-3′.

### 2.11. Antimicrobial Activity Assay

The microbicide property of LHT-8-17 was evaluated by calculating the number of growing microbial colonies in accordance with previously described methods [34,35,36]. Infected wound tissues (2 × 2 cm) from each study group were dissected, placed in Mueller-Hinton broth, and then homogenized for 3 min in homogenizing tubes using multifuctional centrifuge SL8R (Thermo Fisher Scientific, Waltham, MA, USA) to extract the infectious pathogens from infected wound tissues. The supernatant was diluted, inoculated, and cultivated in mannitol salt agar (HiMedia Laboratories Pvt. Limited, Mumbai, India) for 24–48 h at 37 °C. The number of growing colonies of *S. aureus* was calculated per 1 g of the cultivating medium (CFU/g).

### 2.12. Statistical Analysis

Statistical processing of data obtained was carried out using SPSS (version 16.0, IBM, Armonk, NY, USA). Continuous variables were presented as the median or mean (M) value ± square deviation (SD). The distribution of normality was estimated by one-way analysis of variance (ANOVA). Tukey parametric criterion was used for intergroup comparison.

## 3. Results

### 3.1. LHT-8-17 In Vivo Cell Toxicity

**Figure 1:**
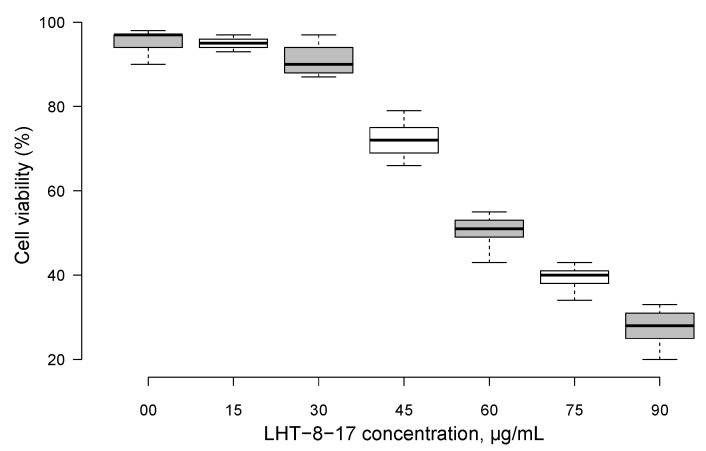
Metabolic activity of skin epidermal cells exposed to different concentrations of cerium-containing N-acethyl-6-aminohexanoic acid compound (LHT-8-17) in MTT assay. Data presented as a percentage of the cell viability in the control, median ± SD.

First, LHT-8-17 toxicity against human epidermal cells was studied (Figure 1). Within concentrations that ranged from 0 to 30 mg/mL, cellular metabolic activity was maintained at about the control level. An increase in the LHT-8-17 concentration in the incubating medium from 30 to 90 µg/mL led to progredient depression of cellular metabolic activity in a concentration-dependent manner.

### 3.2. Efficacy of LHT-8-17 in Healing Linear Wounds

**Figure 2:**
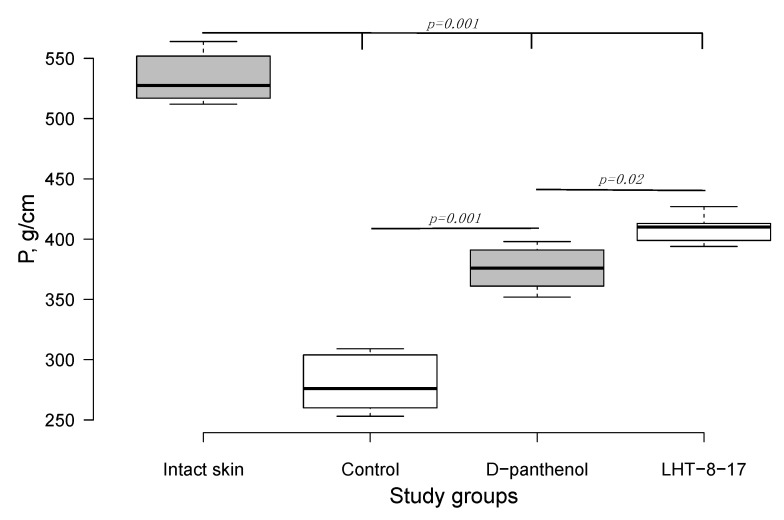
Quantitative evaluation of the skin linear scar firmness in diabetic rats (n = 6 in each group). P (g/cm)—the power needed to rupture the formed scar, presented as the median ± SD. Significant differences were assessed by ANOVA and Tukey tests.

Twenty-one days after the beginning of the experimental treatment of diabetic rat’s skin linear wound, we assessed the efficacy of the cerium-containing formulation. The firmness of each scar was evaluated by registering the power for avulsion of the tissue conjunction formed within the treatment period. As presented in Figure 2, the median power needed to rupture the scar in the control was 283 g/cm; the index value for D-panthenol was 374 g/cm (*p* = 0.001 compared with the control), while in the LHT-8-17 group, it was 415 g/cm (*p* = 0.001 compared with the control and *p* = 0.02 compared with D-panthenol). All scar-related indexes in the study groups were inferior to the value of the power that ruptured intact skin (*p* = 0.001).

### 3.3. Efficacy of LHT-8-17 in Healing Non-Infected Planar Wounds

**Figure 3:**
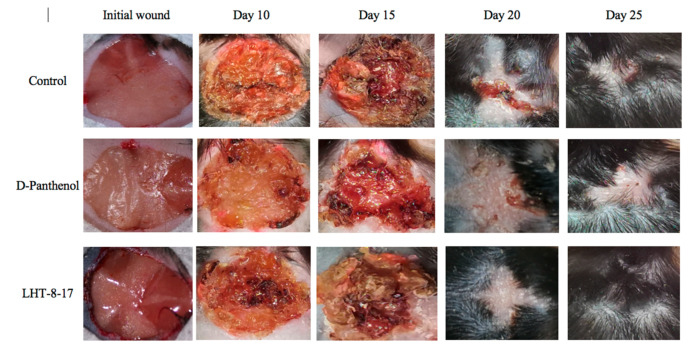
Healing of non-infected planar wounds treated with PBS (control), D-panthenol, and LHT-8-17 for *db*/*db* mice treated with T2D.

We then assessed the healing efficacy of LHT-8-17 based on the model of the experimental non-infected planar wound in T1D and T2D mice. Figure 3 and Table 1 show a dynamic wound area decrease in the study groups. The duration regarding the wound area with a full reduction in control mice with T1D averaged at 23.7 ± 1.3 days, whereas in mice treated with D-panthenol spray and LHT-8-17 spray, it ranged from 17.8 ± 1.1 to 16.3 ± 0.7 days, respectively (*p* < 0.05 in comparison with the control). We also observed the largest scar area, which was calipered on the day of complete wound closure in the control rather than in the treatment groups (*p* < 0.05).

Full self-covering of planar skin defect in control mice with T2D was completed in 28.5 ± 1.5 days and left a 74 ± 4 mm^2^ scar. Both values surpassed similar ones in the case of the T1D control. Local application of 4 mg D-panthenol twice daily for 20 days did not accelerate the wound covering, but resulted in a reduction of the scar area from 74 ± 4 mm^2^ to 56 ± 3 mm^2^ (*p* = 0.005). In contrast, spraying of LHT-8-17 at an equal dose accelerated wound healing to 22.3 ± 1.0 days (*p* = 0.01 in comparison with both the control and D-panthenol) and decreased the scar area.

**Figure 4:**
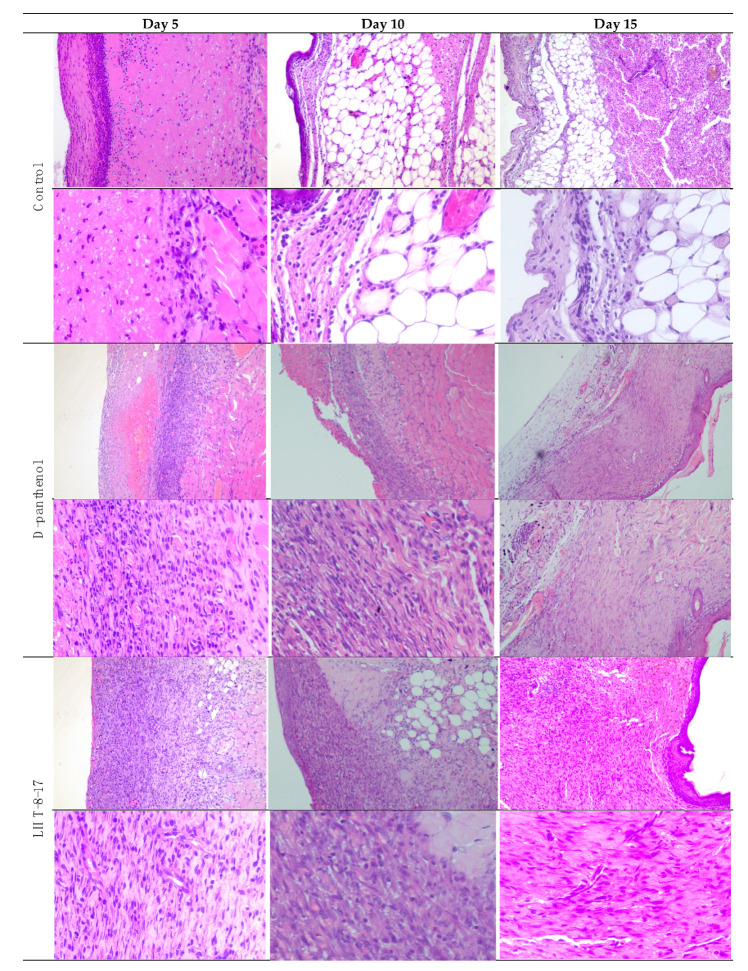
Micromorphology of repairing diabetic wound treated with LHT-8-17, *db*/*db* mice: hematoxylin and eosin, first line in each section—100×, second line in each section—400× (n = 5 in each subgroup). In control: on day 5, the wound bottom is covered by thick dense fibrin with many neutrophils in deep layers (black arrow) while the underlying layer has proliferating fibroblasts (red arrow); on day 10, the wound base is covered by immature granulation tissue, with a superficial thin layer of loose fibrin highly infiltrated by neutrophils (black arrow); underlying deep layers are infiltrated by macrophages, mast cells, lymphocytes, and fibroblasts involving subcutaneous fat tissue (red arrow); on day 15, the wound base central part is covered by fibrin threads, and slight epithelialization is developing on the periphery (black arrow); a thin layer of granulation tissue consists of macrophages, lymphocytes, and some neutrophils (red arrow). In the D-panthenol group: on day 5, thick fibrin with leukocytes covers the wound base, and an underlying thick layer of granulation tissue extends to the fascia propria (black arrow); granulation tissue contains macrophages and lymphocytes, parallel oriented oval and spindle shaped fibroblasts, numerous engorged capillaries (red arrow); on day 10, the wound base is covered by fibrin threads with underlying immature granulation tissue characterized by cell infiltrate and numerous engorged capillaries, some of which have a vertical orientation, oval and spindle shaped fibroblasts (black arrow); diffuse neutrophilic and lympho-macrophage infiltration is noted (red arrow); on day 15, the wound epithelization is developed (black arrow), and the underlying dermis contain mature collagen fibers (red arrow). In the LHT-8-17 group: thin fibrin with leukocytes covers the wound base, and an underlying thick layer of granulation tissue extends to subcutaneous fat tissue (black arrow); granulation tissue consists of cell infiltrate (macrophages, lymphocytes, and neutrophils) and numerous engorged vertically oriented capillaries, as well as parallel-oriented oval and spindle shaped fibroblasts (red arrow); on day 10, epithelization is developing on the wound periphery, and mature granulation tissue is characterized by cell infiltrate, numerous vertically oriented engorged capillaries, and parallel oriented spindle shaped fibroblasts (black arrow); diffuse light neutrophilic and lympho-macrophage infiltration is noted (red arrow); on day 15, the wound epithelization is developed (black arrow), and the underlying dermis contains mature collagen fibers (red arrow).

**Figure 5:**
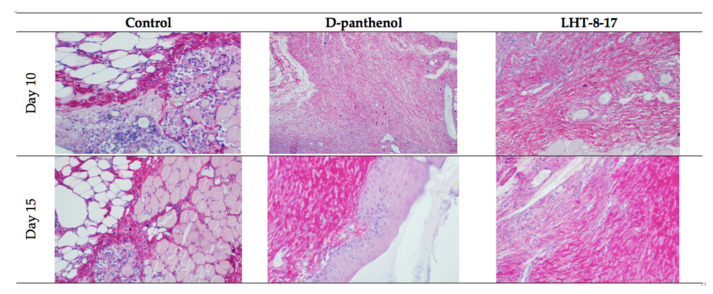
Van Geison’s staining of diabetic wound sections on days 10 and 15 of experimental topical therapy with LHT-8-17, *db*/*db* mice, 400× (n = 5 in each subgroup). Arrows point out positively-stained collagen. Thin and chaotically oriented collagen fascicles intrude muscular and fat layers in the control. Topical application of D-panthenol leads to the formation of a compact collagen layer. In the LHT-8–17 group on day 10, big masses of collagen fascicles form a single layer that transforms to a fibrotic condition by day 15.

Comparative and dynamic evaluation of wound histology showed sufficient differences among study groups dependent on the diabetic model (Figure 4). Thus, wound healing in the control with acute streptozotocin-induced DM was morphologically presented as an inflammation in the non-infected skin defect with edema, lymphoid, and histiocyte tissue infiltration on days 5–10 with the formation of coarse connective tissue scar by day 25 as an outcome. Van Geison’s staining revealed massive collagen expansion as a focus of aseptic inflammation from day 10 of the pathology onset (Figure 5). Both treatment options allowed the prevention of the inflammatory transformation of the process; instead, we observed primary epithelization of the skin defect by day 15 with low intensity of collagen production. In a study with chronic T2D, wound histology of control animals evolved the same way as that in the case of T1D but more slowly (Figure 2, Table 1, Table A1). Topical application of LHT-8-17 induced leukocyte prevalence in the inflammatory focus with more rapid clearance of necrotic debris from the wound. A high intensity of collagen production was observed from day 10. The forming scar was more fragile in comparison with both the control and reference pharmacological agent (Table A1).

**Figure 6:**
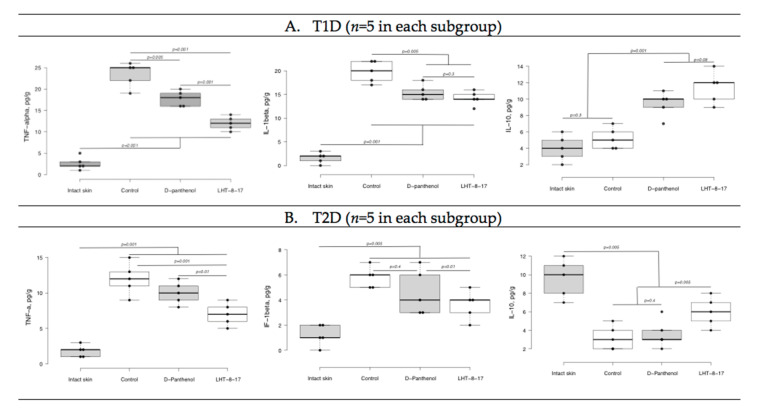
TNF-α, IL-1β, and IL-10 tissue levels on day 5 of wound reparation under topical treatment with LHT-8-17 and the reference medication. Significance of differences was estimated using ANOVA and the Tukey criterion. (**A**) animals with type 1 of DM; (**B**) animals with type 2 of DM.

We then assessed whether LHT-8-17 targeted the intensity of wound inflammation in diabetic animals by determining TNF-α, IL-1β, and IL-10 tissue concentrations (Figure 6). Tissue levels of both pro-inflammatory and resolutery cytokines were determined at the same time point due to sufficient extension of the inflammatory phase of wound healing in diabetic mice accompanied by overregulation of pro-inflammatory signaling [6]. In the model of T1D (Figure 6A), by day 5 of the experiment, the TNF-α concentration in wound tissue increased to 25 ± 4 pg/g (*p* = 0.001 in comparison with intact skin). We measured congruous elevation of the IL-1β tissue level accompanied by depression of the IL-10 concentration (*p* = 0.001). In the group of LHT-8-17-treated animals, the TNF-α tissue concentration averaged 12 ± 1 pg/g, the IL-1β level did not exceed 15 ± 2 pg/g, while the IL-10 concentration reached 12 ± 2 pg/g.

In the control group of animals with type 2 diabetes, both pro-inflammatory cytokine tissue levels were inferior to that of the T1D control group (Figure 6B). While D-panthenol did not address the TNF-α, IL-1β, and IL-10 imbalance, LHT-8-17 partly prevented TNF-α and IL-1β elevation (*p* = 0.001 compared with the control) and IL-10 depression (*p* = 0.005 compared with the control and D-panthenol).

We then evaluated the oxidative status of wound tissues on days 5 and 10 (Table 2) using Fe-induced chemiluminescence. First, wound tissue oxidative status was assessed in the T1D model. On day 5, the LOSA level in wound tissue increased more than twice compared with intact skin (Table 2) whereas TAC proportionally decreased. By day 10, the LOSA level gradually decreased to 6.7 ± 0.3 imp/s, and TAC increased to 1.7 ± 0.2 imp/s. Total application of D-panthenol impacted oxidative stress intensity at both checking points (*p* < 0.05 compared with the control), but had no influence on the tissue antioxidant capacity. In contrast, spraying of 4 mg LHT-8-17 twice daily along with tissue LOSA suppression led to significant TAC elevation up to 2.1 ± 0.3 imp/s (*p* < 0.05 compared with the control) on day 5 and then to 2.7 ± 0.2 imp/s on day 10 (*p* < 0.05 compared with the control and reference drug).

The LOSA level in wound tissue of T2D *db*/*db* mice averaged 12.7 ± 0.4 imp/s on day 5 of the observation and remained elevated by day 10. It was associated with deep depression of TAC. The reference drug partly prevented LOSA growth on days 5 and 10 (*p* < 0.05 compared with the control) and induced TAC on both days of the observation. In the LHT-8-17 group, we registered proportional depression of tissue LOSA accompanied by TAC induction. The changes were similar to the D-panthenol group, but more pronounced on day 10 of the experiment.

**Figure 7:**
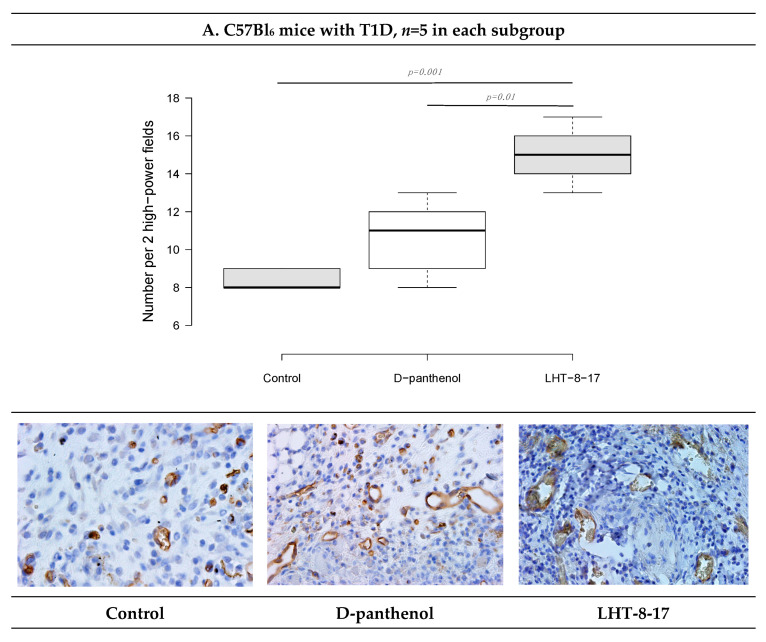

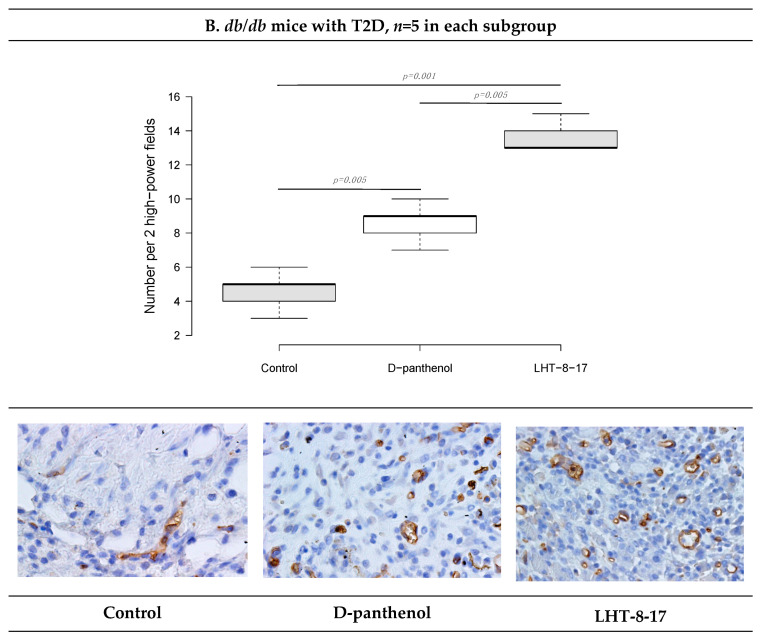
Vascular density and CD34+ expression status in wound tissues of animals with T1D and T2D topically treated with 4 mg LHT-8-17 twice daily, on day 10 of the observation: (**A**,**B**)—T1D and T2D models; micro-vessel number per specimen is presented as the median ± SD, significance of differences was estimated using ANOVA and the Tukey criterion; wound tissue sections stained by rabbit monoclonal anti-CD34 antibody, IHC, 400×.

We then assessed the vascular density of the repairing diabetic wound on day 5 of the experiment onset. It was previously demostrated that endothelial cells expressed CD34; hence, to assess whether LHT-8-17 influenced micro-vessels’ intervention into the wound bed, two randomly chosen fields of tissue section stained by anti-CD34 antibody were evaluated (Figure 7). Topical application of LHT-8-17 stimulated vascularization of newly-developing tissues in the wound bed, and number of micro-vessels in the study group was 14.9 ± 0.7 per two high-power fields vs. 8.3 ± 0.3 in the T1D control (*p* = 0.001). In the model of type 2 diabetes mellitus, the depression of angiogenesis in the control group was even deeper (4.2 ± 0.2), while in the study group, the index averaged at 13.7 ± 0.3 (*p* = 0.001 compared with the control).

**Figure 8:**
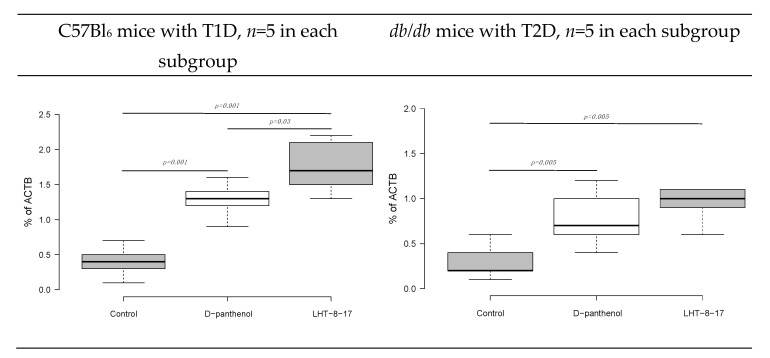
*FGFR3* gene transcript expression as a percentage of the *ACTB* housekeeping gene mRNA concentration in fresh granulations on the day of experimental treatment with LHT-8-17. Data are presented as the median ± SD; significance of differences was estimated using ANOVA and the Tukey criterion.

Fibroblast intervention in the wound bottom reflects the velocity of fresh granulation development. The process is regulated by FGF/FGFR signaling [37]. We then assessed the *FGFR3* gene transcript expression as a percentage of the *ACTB* housekeeping gene mRNA concentration. The marker expression was evaluated in fresh granulations that infiltrated the wound bed on day 10 of the experimental therapy with LHT-8-17 (Figure 8). Connective tissue of both types of diabetic wounds was characterized by low *FGFR3* gene expression by day 10 of reparation with the greatest depression in wounded *db*/*db* mice. Topical treatment with cerium N-acetyl-6-aminohexanoate spray led to significant elevation of the expression up to 1.73 ± 0.4% in the model of T1D (*p* = 0.001 compared with the control), and to 1.05 ± 0.2% in the T2D model (*p* = 0.005 compared with the control).

**Figure 9:**
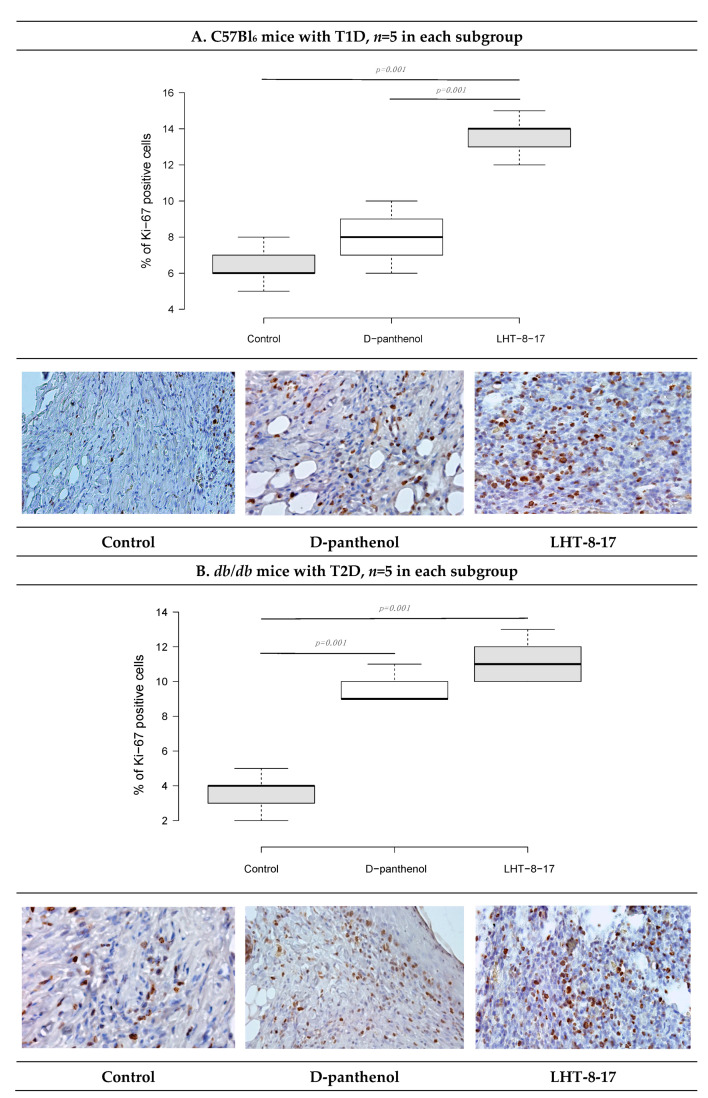
Cellular Ki-67 expression status in wound tissues of animals with T1D and T2D topically treated with 4 mg cerium-containing formulation twice daily, on day 10 of the observation: (**A**,**B**)—T1D and T2D models; quantitative estimation of Ki67 cell positivity presented as the median ± SD; significance of differences was estimated using ANOVA and the Tukey criterion; wound tissue sections stained by rabbit monoclonal anti-Ki-67 antibody, IHC, 400×.

As recently shown, cellular Ki-67 positivity correlated with the proliferative potency of tissues during the regenerative stage of wound healing [38]. The influence of LHT-8-17 on the wound bed cell proliferation was assessed by Ki-67 expression on day 10 of the observation (Figure 9). In the T1D control, only 6.7 ± 0.4% of cells that populated the wound bed displayed Ki-67 positivity. Anti-Ki-67-stained cells were presented largely by fibroblasts, while the keratinocyte population contained single stained cells. Local application of the cerium-containing formulation led to increased Ki-67 cell positivity up to 13.9 ± 0.3% (*p* = 0.001 compared with the control), with sufficient input of epidermal progenitors. The proliferation status of wound bed cells in the T2D control was registered at 4.1 ± 0.3%. Both LHT-8-17 and the reference drug induced proliferation of keratinocytes to 11.4 ± 0.3% and 9.2 ± 0.2% (*p* = 0.001 compared with the control) by day 10 of topical treatment.

### 3.4. Antimicrobial Property of LHT-8-17

**Figure 10:**
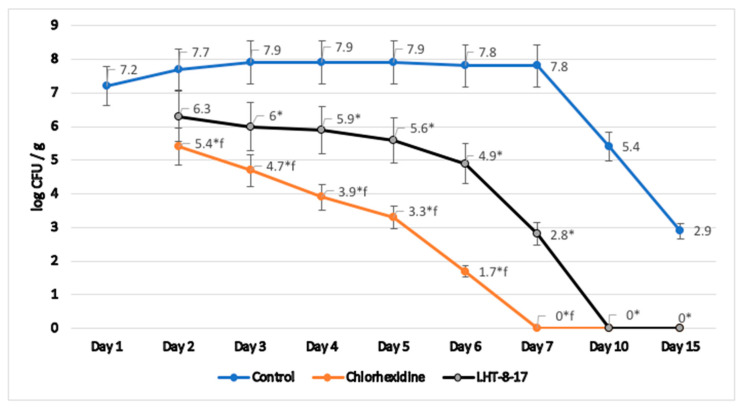
Dynamic *S. aureus* contamination of experimental diabetic wound tissues, T2D *db*/*db* mice; mean ± SD; * *p* < 0.05 compared with the control, ^f^ *p* < 0.05 compared with chlorhexidine (independent *t*-test with Bonferroni’s adjustment), n = 3 at each time point.

Microbicide action of LHT-8-17 was assessed in homogenates of tissues taken from previously infected and topically treated wounds in type 2 diabetic mice. We analyzed the log of number of *S. aureus* colonies per g of specific medium during wound reparation (Figure 10). In the control group, wound contamination decreased from 7.2 (log CFU/g) at the outbreak of the experimental pathology to 2.9 by day 15 of observation. Reference topical antiseptic chlorhexidine depressed *S. aureus* colonization from day 2 of therapy, fully eradicating the wound by day 7 of the observation. LHT-8-17 significantly decreased tissue contamination from day 3 of topical application. Full antimicrobial effect was reached by day 10 of the experimental treatment.

## 4. Discussion

Wound healing is a complex process that requires distinct interaction of multiple subcellular, cellular, and tissue mechanisms. Any negative internal or external impact may disturb the due course of wound reparation, resulting in adverse outcomes such as a delay in cutaneous integrity amendment, the forming of coarse or mutilating scars, development of infectious complications or even septic conditions. Diabetes mellitus occurs throughout the evolution of overlapping phases of hemostasis, inflammatory, regeneration, and epithelization, therefore increasing the severity of the wound condition and adding an additional burden to healthcare institutions.

Topical application of different medications plays one of the key roles in wound reparation rapidity and success. Accelerating wound healing may be associated with antimicrobial, softening, antioxidant, regenerative, and other properties of local drug influence. Taking into consideration the spectrum of pharmacological properties of one of the lanthanoids, cerium, we developed a novel water-soluble cerium-containing molecule with the laboratory name LHT-8-17, using a moiety of organic N-acethyl-6-aminoxecanoic acid for the synthesis. In vitro assessment of LHT-8-17 substance toxicology demonstrated low cellular toxicity in epidermal cell culture, appropriate for topical formulations [39]. Water soluble spray as an appropriate delivery form was chosen for topical therapy of the experimental wound. The study design included comparative evaluation of differences in wound-healing depending on the DM nature; thereby, two methods of the pathology modelling were implemented. Both diabetic conditions are characterized by a stable, high glucose level, but the metabolic profile and cellular and tissue signaling of the experimental disease and response to specific treatment significantly differed depending on its nature [40,41]. It was of great interest for us to determine some particularities that may influence the wound healing process generally and under experimental treatment with LHT-8-17. Insulin-deficient type 1 DM was reproduced in rats and C57BL_6_ mice by streptozotocin administration, whereas *db*/*db* mutant mice of the C57BLKS/J strain served as an in vivo type 2 diabetes biological platform. The T2D model C57BLKS/J genetic background was chosen due to chronic hyperglycemia and dyslipidemia, as well as human-like course of the disease development and progression [42].

Forming scar maturity is one of the objective criteria of wound healing. Among many physical, histological, and biochemical approaches to assessment of the scar maturity, we chose the mechanical one as a direct and reliable method to evaluate scar firmness in an animal study [43,44]. Initial assessment of local 8 mg LHT-8-17 daily application to linear full-layer skin defect in T1D rats demonstrated a 1.46-fold increase of forming scar firmness in comparison with the sham group. In the model of the plain wound, topical therapy with LHT-8-17 spray accelerated wound healing and improved the cosmetic characteristics of the forming scar in mice with both types of DM, thereby surpassing the reference drug’s cure efficacy. To explore the tissue mechanisms of LHT-8-17, local activity wound histology was evaluated. The particularities of tissue reactions in the diabetic wound bed have been broadly studied and published [45,46]. We demonstrated that cerium N-acetyl-6-aminohexanoate, unlike the controls, curbed destructive extension of inflammation in both diabetic models, which was accompanied by early attraction of highly regenerating fresh connective tissues, micro-vascular development, and further epithelization. Comparative evaluation of the tissue reaction between the diabetic models showed deep severity of inflammatory-mediated destruction in the wound bed of T2D mice. One of the pivotal points in recognition of inflammatory process development is quantification of immune cells involved in tissue reactions. Unfortunately, hematoxylin-eosin-stained sections provide only a conditional opportunity to assess the immune cell population; hence, we consider the issue as our study limitation. Separate immune subpopulations will be evaluated by IHC in our further work.

One of the distinctive features of cutaneous reparation in *db*/*db* diabetic mice is also depressed angiogenesis and weak regenerative potency of slowly-forming granulations. A possible explanation of topical LHT-8-17 anti-inflammatory activity may be found in the depression of cytokine TNF-α and IL-1β tissue levels, evaluated on day 5 of the experimental pathology. In turn, a high cytokine tissue level is currently considered an indirect sign of the M1 pro-inflammatory phenotype of macrophages, cells that play a crucial role in wound reparation [47,48,49]. Elevation of the IL-10 level in wound fresh granulations, especially in T1D mice, may indicate a switch to the M2-polarized macrophage phenotype in response to LHT-8-17 topical application [50]. At the same time, further investigation of the macrophage phenotype should be carried out to determine distinct points of cerium-containing formulation influence.

Upregulation of oxidative stress induces cellular damage throughout at least the early phase of the wound process [41,42,43,44,45,46,47,48,49,50,51,52,53]. Recent studies demonstrated the high potency of cerium oxide-containing nanoparticles to abrogate oxidative reactions in biological tissues largely due to the metal variable valence [15]. The effect was explained by superoxide dismutase activation and scavenging reactive oxygen and nitrogen species, both in vivo and in vitro [13,15]. Topical application of the studied spray restrained local oxidative stress reactions on days 5 and 10 of the observation along with the induction of the antioxidant capacity of freshly-growing connective tissues especially in the type 2 diabetes model on day 10 of the experimental therapy. Demonstrated results were forecasted at the design stage of LHT-8-17 development because it is well known that cerium and N-acetyl-6-aminohexanoic acid possess powerful antioxidant activity [15,18]. Vascular density promotes tissue regeneration and therefore supports the accomplishment of wound reparation [54]. We counted micro-vessels in tissues, which penetrated the wound bed, using CD34 membrane positivity of endothelial cells [55]. Topical administration of LHT-8-17-containing spray induced micro-angiogenesis that was critically depressed in both controls. The molecular background of the effect needs further research including the involvement of vascular growth factors, and a possible link between macrophage polarization and vascular growth may serve as a possible explanation [48,49].

Cell proliferation plays a pivotal role in cutaneous defect repair. Lack of the regularity of tissue regeneration in diabetic conditions leads to incompletion wound covering and sustaining of the wound process [8]. Maintaining appropriate tissue recovery is a favorable feature of a promising medication to cure diabetic wounds. Topical use of the LHT-8-17 spray propelled proliferative potency of fibroblasts and pro-keratinocytes. This was accompanied by upregulation of *FGFR3* gene expression on day 10 of the observation [56]. Both effects were more pronounced in the streptozotocin-induced model of DM.

A high rate of microbial contamination of diabetic skin defects by nosocomial bacteria such as methicillin-resistant *S. aureus* and *P. aeruginosa* requires adequate wound anti-infectious control [9]. The microbicidal potency of cerium along with the anti-viral action of N-acetyl-6-aminohexanoic acid has been previously reported [11,18]. Stepanenko et al. showed that cerium-containing N-acetyl-6-aminohexanoate prevented colony growth of meticillin-resistant *S. aureus* in vitro [57]. We used an approach for anti-microbial activity determination, which allowed us to detect bacteria not only in washouts but in all wound tissues [34,35,36]. LHT-8-17 eradicated the wound bed from methicillin-resistant *S. aureus* strain АТСС 43300 by day 10 of its topical application (4 mg) twice daily. The dynamic of the defect anti-microbial cleaning was the same as that for the reference antiseptic agent chlorhexidine.

## 5. Conclusions

Cerium-containing N-acetyl-6-aminohexanoic acid formulation as a 10 mg/mL spray demonstrated promising wound-healing activity in experimental models of type 1 and type 2 diabetes mellitus. Acceleration of cutaneous defect covering was a result of multiple biological properties of the novel molecule, such as optimization of regulatory cytokine tissue level, promotion of tissue regeneration and vascularization, and curbing of local oxidative stress reaction and anti-*S. aureus* activity. The obtained results allow us to consider the formulation as a promising pharmacological agent for diabetic wound topical treatment. At the same time, the macrophage phenotype characteristics and their link with the expression of major growth factors will be in the focus of our further study for more precise determination of the LHT-8-17 cellular and molecular mechanisms of action in diabetes animal models.

## Figures and Tables

**Table 1:** The dynamics of non-infected wound areas (% of initial area’s square) in streptozotocin-induced (T1D) and *db/db* (T2D) mice, topically treated with 4 mg LHT-8-17 and 4 mg D-panthenol as a spray twice daily, mean ± SD.

Study Group	Wound Area, % of Initial Area’s Square						Average Time of Full Wound Healing, Days	Scar Area *, mm^2^
	Initial Wound	Day 10	Day 15	Day 20	Day 25	Day 30		
C57Bl_6_ mice with T1D								
Control	n = 20 100 ± 0	n = 15 55 ± 2	n = 10 32 ± 3	n = 5 9 ± 4	n = 5 0 ± 0	n = 5 0 ± 0	23.7 ± 1.3	56 ± 4
D-Panthenol	n = 20 100 ± 0	n = 15 25 ± 3 ^†^	n = 10 6 ± 4 ^†^	n = 5 0 ± 0 ^†^	n = 5 0 ± 0	n = 5 0 ± 0	17.8 ± 1.1 ^†^	39 ± 3 ^†^
LHT-8-17	n = 20 100 ± 0	n = 15 21 ± 4 ^†^	n = 10 5 ± 3 ^†^	n = 5 0 ± 0 ^†^	n = 5 0 ± 0	n = 5 0 ± 0	16.3 ± 0.7 ^†^	39 ± 4 ^†^
*db*/*db* mice with T2D								
Control	n = 20 100 ± 0	n = 15 70 ± 4	n = 10 42 ± 5	n = 5 13 ± 4	n = 5 7 ± 3	n = 5 0 ± 0	28.5 ± 1.5	74 ± 4
D-Panthenol	n = 20 100 ± 0	n = 15 76 ± 3	n = 10 35 ± 4	n = 5 16 ± 3	n = 5 5 ± 3	n = 5 0 ± 0	27.7 ± 1.1	56 ± 3 ^†^
LHT-8-17	n = 20 100 ± 0	n = 15 33 ± 4 ^†,‡^	n = 10 21 ± 3 ^†,‡^	n = 5 5 ± 2 ^†,‡^	n = 5 0 ± 0 ^†,‡^	n = 5 0 ± 0	22.3 ± 1.0 ^†,‡^	43 ± 4 ^†,‡^

Note*:* ^†^ *p* < 0.05 compared with the control group, ^‡^ *p* < 0.05 compared with D-panthenol (ANOVA and Tukey criterion); * scar area was calipered on the day of complete wound closure.

**Table 2:** Local oxidative stress activity (LOSA) and total antioxidant capacity (TAC) of wound tissue of diabetic mice topically treated with LHT-8-17.

Study Group	Fe-Induced Chemiluminescence, ×10^3^ imp/s			
	Day 5		Day 10	
	LOSA	TAC	LOSA	TAC
T1D model (*n* = 5 in each subgroup)				
Intact skin	2.7 ± 0.3	3.3 ± 0.2	3.0 ± 0.2	3.1 ± 0.3
Control	8.3 ± 0.4 *	1.0 ± 0.2 *	6.7 ± 0.3 *	1.7 ± 0.2 *
D-panthenol	6.6 ± 0.3 *^,#^	1.7 ± 0.4 *	4.3 ± 0.4 *^,#^	1.9 ± 0.3 *
LHT-8-17	5.2 ± 0.2 *^,#^	2.1 ± 0.3 *^,#^	4.2 ± 0.3 *^,#^	2.7 ± 0.2 ^#,†^
T2D model (*n* = 5 in each subgroup)				
Intact skin	3.1 ± 0.3	2.9 ± 0.4	3.7 ± 0.3	2.4 ± 0.4
Control	12.7 ± 0.4 *	0.5 ± 0.1 *	11.8 ± 0.5 *	0.7 ± 0.2 *
D-panthenol	9.4 ± 0.3 *^,#^	1.3 ± 0.3 *^,#^	8.9 ± 0.4 *	1.5 ± 0.3 ^#^
LHT-8-17	8.3 ± 0.5 *^,#^	1.9 ± 0.2 *^,#^	6.3 ± 0.3 *^,#,†^	2.2 ± 0.1 ^#,†^

Note: *p* < 0.05 * compared with intact skin; ^#^ compared with appropriate control; ^†^ compared with D-panthenol (ANOVA; Tukey criterion).

## Data Availability

The data presented in this study are available on request from the corresponding author. The data are not publicly available due to institutional restrictions.

## Appendix A

**Table A1:** Histological scoring of T2D diabetic wounds topically treated with LHT-8-17 at different timepoints of the healing process according to [32].

Day of the Observation	Study Group	Exudation	Presence of Bacterial Colonies	Inflammatory Infiltration	Microcirculation Disorder	Proliferation of Fibroblasts	Neo-Angiogenesis	Prevalence of Granulation Tissue	Maturity of the Granulation Tissue	Fibrous Tissue
5	D-panthenol	2	0	3	2	1	3	2	2	0
	LHT-8-17	1	0	2	1	1	3	3	3	0
	Control	3	0	3	3	0	0	0	0	0
10	D-panthenol		0	1	0	3	3	3	3	3
	LHT-8-17		0	1	0	3	3	3	3	3
	Control	2	0	3	3	2	1	2	1	1
15	D-panthenol	0	0	0	1	0	3	3	4	3
	LHT-8-17	0	0	0	1	0	3	3	4	3
	Control	1	0	0	1	0	1	1	1	1

Note: 0—absence of a feature, 1—mild, 2—moderate, 3—high/severe level of a histological feature/damage development.
